# LiraSearch—ultrafast ligand shape and electrostatic matching server

**DOI:** 10.1093/bioadv/vbag139

**Published:** 2026-05-18

**Authors:** Rinaldo W Montalvão, Simon Bray, Marcos Veríssimo-Alves, Elena Cubero, Björn Grüning, Vitor B Pinheiro

**Affiliations:** Gain Therapeutics Sucursal España, Gain Therapeutics, Barcelona Science Park, Barcelona, 08028, Spain; Gain Therapeutics Inc., Gain Therapeutics, Bethesda, MD 20814, USA; Physics Dept. - ICEx, Universidade Federal Fluminense, R. Des. Ellis Hermydio Figueira, Volta Redonda, RJ 27213-145, Brazil; Gain Therapeutics Sucursal España, Gain Therapeutics, Barcelona Science Park, Barcelona, 08028, Spain; Bioinformatics Group, Department of Computer Science, University of Freiburg, Freiburg, 79110, Germany; Rega Institute for Medical Research, KU Leuven, Leuven, Flemish Brabant 3000, Belgium

## Abstract

**Summary:**

Spherical harmonics have emerged as a powerful framework for molecular shape and electrostatic comparison in ligand-based virtual screening. Here, we present LiraSearch, a high-performance implementation and web server that uses spherical harmonics expansions to encode and compare three-dimensional (3D) molecular shapes and electrostatic potential (ESP) surfaces of drug-like compounds. Molecular surfaces are transformed into compact, low-dimensional spherical harmonics descriptors that capture both geometric and electrostatic features. These descriptors enable rapid similarity calculations between query molecules and large compound libraries while exploiting the rotational invariance of spherical harmonics to avoid explicit alignment. The representation is robust to moderate conformational variation and is effective at identifying structurally diverse active compounds sharing similar pharmacophoric patterns, facilitating scaffold hopping in ligand discovery workflows. Previous studies have shown that spherical harmonic surface representations achieve high retrieval performance with relatively low expansion orders and compact descriptors. By integrating these representations into an efficient screening pipeline, LiraSearch enables rapid similarity searches across ultralarge chemical libraries while preserving both geometric and electrostatic fidelity. This framework provides a scalable, mathematically rigorous approach to ligand similarity searching and can support early-stage hit discovery as well as downstream applications, such as protein–ligand docking and structure-based pharmacophore modelling.

**Availability and implementation:**

LiraSearch is a set of open-source programs developed in C/C++, Python and Julia. The source code is available at https://github.com/gaintherapeutics/LiraSearch, and the server is at https://usegalaxy.eu/? tool\_id=lirasearch.

## 1 Introduction

Small-molecule compounds have always played a central role in drug discovery. Whether derived from natural products, structural analogues, or synthetic leads, they form the cornerstone of therapeutic development (Drews 2000; Li and Vederas 2009). This success has been bolstered by increased investment and the adoption of new technologies that enhance both candidate throughput and data quality, such as high-resolution protein structures. More recently, computational approaches, particularly machine learning (ML) and artificial intelligence (AI), have accelerated screening, molecular design, and automation (Schneider 2018).

High-throughput screening (HTS) programs aim to rapidly test large compound libraries to identify hits with IC_50_ values below 10 μM. Achieving this requires scalable compound synthesis, combinatorial chemistry, and automated bioassays. However, despite significant investments, the rate at which clinical trial candidates are identified has not increased proportionally.

Targeted approaches focus on tractable protein families, guided by structural data and known ligands, enabling the creation of focused libraries for families such as kinases and proteases (Irving *et al.* 2001). Fragment-based screening, using small molecules (100–250 Da), identifies leads with high ligand efficiency through biophysical methods such as NMR and X-ray crystallography (Blundell *et al.* 2006; Li 2020). Although these fragments often bind weakly, they provide excellent starting points for lead optimization. However, fragment-based strategies are resource-intensive, particularly when structural determination proves difficult.

The advent of advanced AI tools such as AlphaFold 3 makes the prospect of purely *in silico* drug discovery pipelines increasingly feasible. However, human input remains essential at many steps, and more autonomous AI systems remain needed in drug discovery.

A critical component of automated drug discovery is the analysis of ligand similarity. Traditional ligand-based virtual screening approaches, such as ultra-fast shape recognition (USR) (Ballester and Richards 2007) and USRCAT (Schreyer and Blundell 2012), provide efficient shape-based comparisons and have been widely adopted for the rapid screening of large compound libraries. Methods like ElectroShape and eSim (Armstrong *et al.* 2010; Cleves *et al.* 2019) extend these capabilities by incorporating simplified electrostatic models. However, these approaches are limited in several ways: they often lack quantum-mechanical accuracy in their treatment of electrostatics, and their computational frameworks are not fully unified, making it difficult to integrate both shape and electrostatic features seamlessly. Moreover, existing methods may struggle to capture the full complexity of molecular recognition, particularly the influence of localized charge distributions, and can become computationally intensive when scaling to ultralarge chemical libraries ($10^{9}-10^{12}$ compounds). These limitations highlight the need for a more robust, scalable, and accurate solution for virtual screening.

Recent developments aiming to overcome the limitations in shape-based screening include methods such as UFSRAT Shave *et al.* 2015 and GPU-accelerated approaches like ROSHAMBO2 (Atwi *et al.* 2025), which aim to improve the efficiency of large-scale ligand similarity searches. UFSRAT provides highly efficient ligand-based virtual screening by encoding molecular shape and atom-type information into moment-based descriptors, enabling similarity searches across multi-million-compound libraries in a few seconds. GPU-accelerated approaches such as ROSHAMBO2 enable practical screening of ultralarge chemical libraries by combining Gaussian-based shape and pharmacophore alignment with highly parallel GPU computation, delivering performance improvements exceeding 200-fold over earlier implementations.

Here, we present another approach wich address these limitations, LiraSearch is a server that employs spherical harmonics for molecular surface analysis (Ritchie and Kemp 1999; Morris *et al.* 2005; Mavridis *et al.* 2007). Previous studies have demonstrated that spherical harmonic surface representations provide an efficient framework for molecular shape comparison and virtual screening, achieving high retrieval performance with relatively low expansion orders and compact descriptor representations. LiraSearch combines both shape and electrostatic descriptors into a compact, rotationally invariant representation, enabling highly efficient and accurate comparisons of molecular surfaces. A graph-convolutional neural network (Rathi *et al.* 2020) is used to provide high-fidelity electrostatic potential approximations, eliminating the computational cost typically associated with *ab initio* quantum mechanical calculations. The neural network is trained on a large dataset of high-quality density-functional-theory electrostatic potentials, comprising hundreds of thousands of molecular conformations, and successfully reproduces the electrostatic potentials with high fidelity, achieving correlation coefficients typically above 0.9 and errors of only a few ${\text{kcal·mol}}^{-1}$ in the predicted surface potentials.

This integrated approach is particularly valuable for searching extremely large molecular databases, which can contain millions to billions of compounds. Traditional quantum-based similarity methods are too computationally intensive to be applied at this scale, and shape-only metrics fail to capture key features that govern molecular recognition, such as localized charge distributions. Using spherical harmonics and deep learning, LiraSearch enables rapid, scalable comparisons that take into account both geometry and electrostatics; critical attributes for virtual screening, scaffold hopping, and ligand-based drug design. Moreover, its rotational invariance and compact feature encoding significantly reduce computational overhead, making it well-suited for deployment in cloud-based or distributed screening pipelines where both speed and precision are essential.

## 2 Methods

Spherical harmonics offer a unifying framework for describing mathematical functions defined on the unit sphere. They provide a complete representation and a natural structure for handling rotations (Ritchie and Kemp 1999). These geometric properties make spherical harmonics particularly well-suited for approximating molecular surface shapes and electrostatic potentials, with the added benefits of compactness and ease of comparison. While a surface mesh representation requires hundreds of thousands of data points, the spherical harmonics coefficients consist of only a few hundred. Our approach leverages these features to construct well-defined geometric descriptors directly applicable to virtual screening.

LiraSearch utilises a multilayered framework for comparing molecular surface shapes and electrostatic potentials. All layers share a common mathematical foundation based on the three-dimensional representation of “star-shaped” (i.e., single-valued) functions using spherical harmonics. Specifically, we assume that rays radiating from the molecular centre of geometry intersect the surface only once. This “star-shaped” approximation is generally sufficient for solvent-accessible molecular surfaces and has been widely used in spherical harmonic surface representations (Morris *et al.* 2005). Although highly concave geometries could, in principle, violate this assumption, small drug-like molecules typically exhibit near-convex surface envelopes, making the approximation appropriate for ligand-similarity comparisons. Previous studies have shown that the resulting spherical harmonic descriptors remain robust for molecular ranking and virtual screening applications under these conditions Mavridis *et al.* (2007).

### 2.1 Mathematical approach

Spherical harmonics are an important class of orthogonal functions whose linear combinations can express any real square integrable function of θ and ϕ defined in 3D space. The series expansion of a given function f(θ,ϕ) in spherical harmonics is


(1) $$f\left(\theta ,\phi \right)=\sum_{\ell =0}^{\infty }\sum_{m=-\ell }^{\ell }a_{\ell m}Y_{\ell }^{m}\left(\theta ,\phi \right),$$

Where f(θ,ϕ) is the function to be expanded, $Y_{\ell }^{m}\left(\theta ,\phi \right)$ a particular spherical harmonics, $a_{\ell m}$ its coefficient, θ is the polar angle and ϕ is the azimuthal angle of the spherical coordinate system (ISO 80000–2: 2019 convention), and ℓ and *m* are the degree and order of the spherical harmonics, respectively. They are defined as real values by:


(2) $$\begin{array}{c} Y_{\ell }^{m}\left(\theta ,\phi \right)=\{\begin{array}{cc} {\bar{P}}_{\ell }^{m}\left(\text{cos}\;\theta \;\;\text{cos}\;m\theta \right), & m\geq 0\\ {\bar{P}}_{\ell }^{\left|m\right|}\left(\text{cos}\;\theta \;\;\text{sin}\;\left|m\right|\theta \right), & m<0 \end{array} \end{array}$$

In equation (2), ${\bar{P}}_{\ell m}$ is the normalized associated Legendre polynomial, defined as:


(3) $${\bar{P}}_{\ell }^{m}\left(x\right)=\sqrt{\frac{2\ell +1}{4\pi }\frac{\left(\ell -m\right)!}{\left(\ell +m\right)!}}P_{\ell }^{m}\left(x\right).$$

and, $a_{\ell m}$ can be calculated as:


(4) $$a_{\ell m}=\int_{\Omega }f\left(\theta ,\phi \right)Y_{\ell }^{m}\left(\theta ,\phi \right)d\Omega .$$

where dΩ=sin θdθdϕ is the differential surface area on the unit sphere (Atkinson and Han 2012).

The function f(θ,ϕ) can describe either the radius or the electrostatic potential (ESP) on the surface. The molecular ESP is generated by the graph convolutional deep neural network (DNN) model of Rathi *et al.* (2020), trained on DFT-generated ESP surfaces for 105 500 molecule models.

Equation (1) allows the reconstruction of the surface and its electrostatic potential by truncating the expansion of f(θ,ϕ) at a maximum value of the spherical harmonics degree *L*. This surface shape and electrostatic representation exhibit a coarse-to-fine nature; thus, their details increase with the maximum degree level. A carefully selected *L* creates a small number of coefficients that describe the dual shape-electrostatic information in a very compact form.

For each set of spherical harmonic coefficients (shape and electrostatics), we can define rotational invariant fingerprints (RIF) as described by Mavridis *et al.* (2007):


(5) $$A_{\ell }={\left(\sum_{m=-\ell }^{\ell }a_{lm}^{2}\right)}^{1/2}$$

and,


(6) $$A_{L}={\left(\sum_{\ell =0}^{L}A_{\ell }^{2}\right)}^{1/2}$$

Lastly, the metric for comparing two ligands is given by:


(7) $$D_{\mathrm{RIF}}=A_{L}^{2}+B_{L}^{2}-2\sum_{\ell =0}^{L}A_{\ell }B_{\ell },$$

Distance Rotation-Invariant Fingerprints ($D_{\mathrm{RIF}}$) can be viewed as an Euclidean-like distance applied to a specially constructed, rotation-invariant descriptor vector (RIF), making it suitable for rapid comparison of molecular shapes without explicit alignment. Mavridis *et al.* (2007) show that L=15 yields descriptors with sufficient precision for comparison of ligands.

LiraSearch can also rotate the ligands to align their shapes. The alignment is achieved through the implementation of the Iterative Closest Point (ICP) algorithm (Besl and McKay 1992) by Funkhouser *et al.* (2004) using the SOFT 1.0 spherical harmonics library (Kostelec and Rockmore 2003). This technique determines the Wigner-*D* rotation matrix that aligns the two sets of coefficients by calculating an FFT on the SO(3) Rotation Group. By applying the optimal Wigner-*D* rotation matrix to the coefficients of one of the functions, the algorithm aligns the two shapes in an orientation that enables direct comparison. Additional details concerning the molecular surface representation, the electrostatic model and numerical calculation of the spherical harmonics coefficients are discussed in Section 1, available as supplementary material at *Bioinformatics Advances* online of the supplementary material.

## 3 Implementation

The components of LiraSearch use a combination of programming languages to meet the various demands of its complex systems. The primary scripts are written in Python for flexibility and easy maintenance. The computation of spherical harmonic components is handled in Julia, while the ligand superposition program is developed in C++. Julia and C++ allow these components to effectively leverage the multiple cores of CPUs or GPUs, delivering performance that meets the needs for ligand searching in ultra-large datasets.

**Figure 1:**
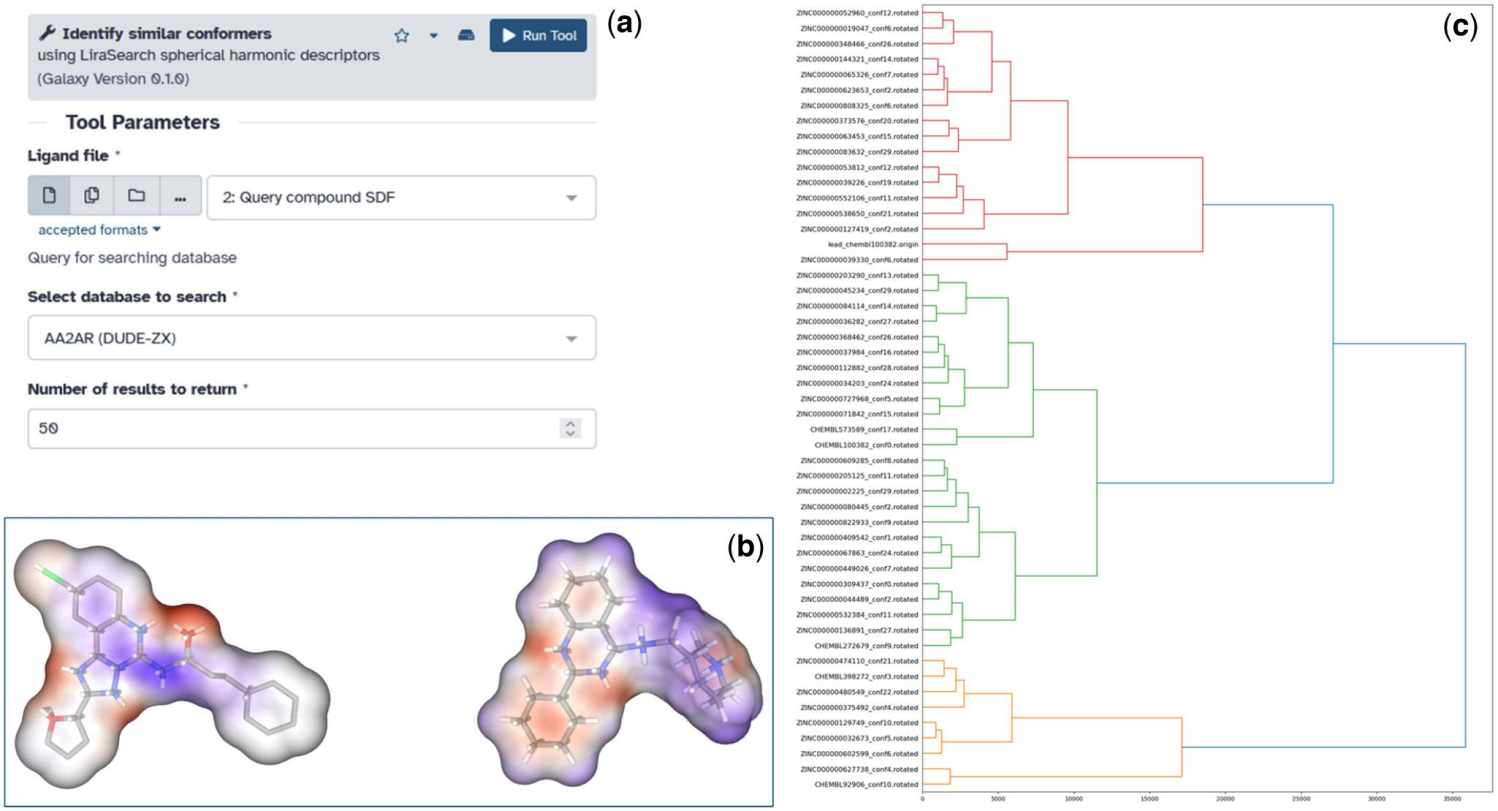
LiraSearch workflow and analysis outputs. (a) Screenshot of the LiraSearch web server interface implemented in Galaxy, showing its key input parameters. Users can upload a query compound in SDF format, select from curated databases, and specify the number of similar conformers to retrieve. The streamlined user interface facilitates rapid virtual screening of large chemical libraries using spherical harmonic descriptors for both shape and electrostatic properties. (b) Representative visualisations of molecular electrostatic surface potentials for two query compounds, as generated by the Jupyter notebook accompanying server results. Molecular surfaces are rendered with colour mapping of the electrostatic potential, highlighting regions favourable for ligand-receptor interactions and facilitating assessment of chemical diversity among hits. (c) Hierarchical clustering dendrogram of top-ranked compounds returned by the LiraSearch server in a screening run against the AA2AR (DUDE-Z) family. Branches group conformers by shape and electrostatic similarity, with distinct clusters capturing chemically diverse scaffolds sharing comparable pharmacophoric features. This clustering demonstrates LiraSearch’s utility for scaffold hopping and identification of novel chemotypes beyond close structural analogues.

LiraSearch is provided as a webserver (Fig. 1a), implemented with the Galaxy platform (Galaxy Community, 2024), and is publicly available via the European Galaxy server at https://usegalaxy.eu/? tool\_id=lirasearch. The user uploads a query compound in SDF or PDB format, selecting the database to search and the desired number of results. The user can choose to search several provided databases, including ChEMBL (Gaulton *et al.* 2012), the entire set of published ligands from the Protein Data Bank (Berman *et al.* 2000), and various ligand families from the DUDE-Z dataset (Stein *et al.* 2021). After the execution is complete, the best matches are returned in SDF and PQR formats, and a Jupyter notebook with fingerprint analysis (Figs. 1b and c) is also included. Internally, a surface is computed from the query compound, which is used to calculate the spherical harmonics descriptor. A compound database is searched, and compounds with similar descriptors are retrieved and aligned to the query before being returned to the user. Within the webserver, the entire pipeline runs within a Docker container, which is also made available to users who prefer a command-line interface (https://hub.docker.com/r/sb17/lirasearch/tags).

## 4 Discussion

It is important to note that the goal of LiraSearch is not to introduce a new molecular similarity metric, but to provide a scalable and accessible implementation of the spherical harmonic surface comparison framework. The underlying spherical harmonics-based approach has previously been evaluated in dedicated benchmarking studies that assessed ligand retrieval performance using ROC-based virtual screening experiments and clustering analyses. The present work, therefore, focuses on enabling efficient application of this methodology to modern ultra-large chemical libraries by improving implementation, integrating already available electrostatic descriptors, and deploying it as an open-access web server.

The spherical harmonic surface comparison methodology used in LiraSearch builds directly on the framework described by Mavridis *et al.* (2007), which included extensive benchmarking of ligand similarity using ROC-based retrieval tests and clustering experiments. Those studies demonstrated that relatively low-order spherical harmonics expansions (typically L≈6) provide an effective balance between computational efficiency and screening accuracy, enabling reliable ligand similarity searches and chemically meaningful clustering using compact descriptor representations. In particular, rotational spherical harmonics comparisons were shown to achieve high retrieval performance in virtual screening experiments while maintaining very low computational cost for pairwise comparisons.

Applying spherical harmonics-based clustering to drug and odour molecule datasets, Mavridis *et al.* (2007) demonstrates that it yields chemically meaningful groupings that often match or surpass conventional physicochemical descriptor approaches. Importantly, because spherical harmonics representations are based on shape rather than covalent structure, they enable “scaffold hopping,” helping identify novel chemotypes that traditional similarity searches might miss. Consistent with this, applying LiraSearch on the original dataset and protocol of Mavridis *et al.* (2007) reproduces the reported behaviour with minimal deviation, indicating that the key properties of spherical harmonics-based similarity are preserved in the present implementation.

This shape-based approach is particularly valuable because it captures the global 3D characteristics that govern ligand–receptor interactions, rather than focusing only on close chemical analogues. By enabling rapid comparisons of surface envelopes, spherical harmonics methods can retrieve structurally diverse compounds that share key pharmacophoric features, making them well-suited for early-stage drug discovery, where novelty is essential. Moreover, since the spherical harmonics framework can be extended to encode additional surface properties (e.g., electrostatic potential, hydrophobicity, polarizability), it offers a flexible platform for more refined similarity searches. This combination of efficiency, scalability, and extensibility positions spherical harmonic surface representations as a practical alternative to conventional fingerprinting and more computationally intensive 3D methods in virtual screening workflows (Mavridis *et al.* 2007; Kumar and Zhang 2018).

To ascertain the scalability of this approach, we applied the LiraSearch screening method to an ultra-large chemical library containing 4 billion molecules from the ZINC22 database. The dataset comprises approximately 80 million individual compounds, each associated with about 50 conformers generated using the RDKit ETKDGv3 method and subsequently optimised through MMFF94s energy minimisation (Halgren 1999; Riniker and Landrum 2015). The calculations were performed on a single workstation equipped with an AMD Threadripper Pro 7995WX processor, 256 GB of memory, and an RTX A6000 Ada GPU. Despite the vast size of the search space, the descriptor-based similarity-comparison stage of the screening workflow was completed in approximately 3 hours after spherical-harmonic descriptors had been precomputed for the database molecules. During the search, only the five highest-ranked conformations are reported for each compound.

This corresponds to a screening throughput of approximately $3.7\times 10^{5}$ molecules per second, enabling practical exploration of multi-billion–compound chemical libraries on commodity hardware. For comparison, the widely used UFSRAT (Ultra-Fast Shape Recognition with Atom Types) method reports screening speeds of approximately $4.7\times 10^{5}$ molecules per second in its original benchmark on a database of 3.8 million molecules. Thus, the performance of LiraSearch lies within the same throughput regime as one of the fastest published alignment-free 3D similarity screening approaches, while demonstrating scalability to libraries that are three orders of magnitude larger. These results highlight the computational efficiency of spherical harmonic surface representations for molecular shape encoding. More broadly, the method is well suited for modern drug discovery pipelines, where ultra-large virtual libraries containing billions of compounds are becoming increasingly common. Together, these results demonstrate the practical feasibility of rapid, shape-driven identification of novel ligands at previously inaccessible chemical scales. A full benchmark using the DUDE-Z dataset is reported in Section 2, available as supplementary material at *Bioinformatics Advances* online of the supplementary materials.

## 5 Conclusion

LiraSearch offers several key advantages over existing ligand-based virtual screening tools. Its compact, rotationally invariant descriptors, and efficient implementation enable scalable, high-throughput screening of vast chemical libraries while maintaining both geometric and electrostatic fidelity. The reconstructed spherical harmonic surface representations reproduce the original molecular surface geometry with a root-mean-square deviation (RMSD) of 0.19 ± 0.01 Å, while the reconstructed electrostatic potential values show an RMSD of $2.0\;\pm \;0.6{\text{ kcal·mol}}^{-1}$ relative to the reference electrostatic surface values. The approach remains effective for typical ligand conformations encountered in virtual screening workflows. Furthermore, LiraSearch is well-suited for integration into cloud-based or distributed screening pipelines, making it a valuable resource for both academic and industrial research teams seeking to streamline virtual screening, structure-based pharmacophore modelling, and protein-ligand docking efforts.

## Supplementary Material

vbag139_Supplementary_Data
