# Supplementary material for: LiraSearch—ultrafast ligand shape and electrostatic matching server
Source: Bioinform Adv. 2026 May 18;6(1):vbag139. doi: 10.1093/bioadv/vbag139 (PMC13221974; doi:10.1093/bioadv/vbag139)
Supplement: vbag139_Supplementary_Data [file vbag139_supplementary_data.pdf]

LiraSearch – ultrafast ligand shape and electrostatic matching server: supplemental document

This supplementary document presents additional methodological details and validation experiments supporting the LiraSearch platform for ultrafast ligand-based virtual screening using spherical harmonic (SH) representations of molecular shape and electrostatic potential. The construction of molecular surfaces and electrostatic fields is described, utilizing a graph-convolutional neural network trained on density functional theory (DFT)-derived electrostatic potentials. These fields are then projected onto spherical coordinates and expanded into SH coefficients. The sampling and interpolation procedure includes surface ray casting, barycentric interpolation, and numerical integration over the unit sphere to ensure consistent and reproducible mapping of both geometry and electrostatics. The alignment of SH descriptors is further detailed, employing fast Fourier transforms on the $SO(3)$ rotation group to enable efficient rotational matching without explicit spatial superposition.

1. MOLECULAR SURFACES CONSTRUCTION AND ALIGNMENT

A. Molecular Surface Shape and Electrostatic Potential

In LiraSearch, the function $f(\theta, \phi)$ represents either the radius of a molecular surface (MLS) or its electrostatic potential (ESP) at that radius (fig. S1), with the molecular centre-of-geometry positioned at the origin of the coordinate system. The function $f(\theta, \phi)$ is defined over a triangular mesh, typically comprising approximately 3,000 to 4,000 vertices, that encodes both the MLS and the corresponding ESP at each vertex (fig. S2). This mesh is produced by a graph convolutional deep neural network (DNN) model [1], which is trained on ESP surfaces generated by density functional theory (DFT) for 105,500 molecular models, selected from a pool of 1,336,480 commercially available molecules. A concise description of this process follows.

First, feature groups containing non-hydrogen and hydrogen atoms of a molecule, plus lone pairs, p orbitals, and σ-holes, are generated, each with point charges, for all molecules in the set. Molecular geometries were optimised by DFT at the B3LYP/6-31G* level of theory and ESP surfaces are then generated by single-point calculations at the B3LYP/6- 311G** level of theory. Next, the point charges for the feature groups are fitted to optimally reproduce the DFT ESP surfaces, generating the DFT-fp (fp: feature-point charge) model for point charges. Finally, DFT-fp results are used to train a molecular graph convolutional DNN to generate point-charge values for feature groups of molecules not in the training set.

Although it is feasible to generate a set of spherical harmonic (SH) coefficients from a DNN for direct reconstruction of the DFT ESP, the ESP-DNN developed by Rathi *et al.* [1] was selected for two main reasons. First, the ESP surfaces produced by the pre-trained DNN from Rathi *et al.* are comparable in quality to those obtained from the full DFT calculations. Second, the DFT-fp calculation requires 0.3 seconds, making it approximately 6,000 times faster than the DFT calculation. As the ESP-DNN used remains among the most accurate and fastest to compute, its implementation is justified. The source code for the DNN is available at https://github.com/AstexUK/ESP_DNN, and for the mesh generator at https://github.com/AstexUK/esp-surface-generator.

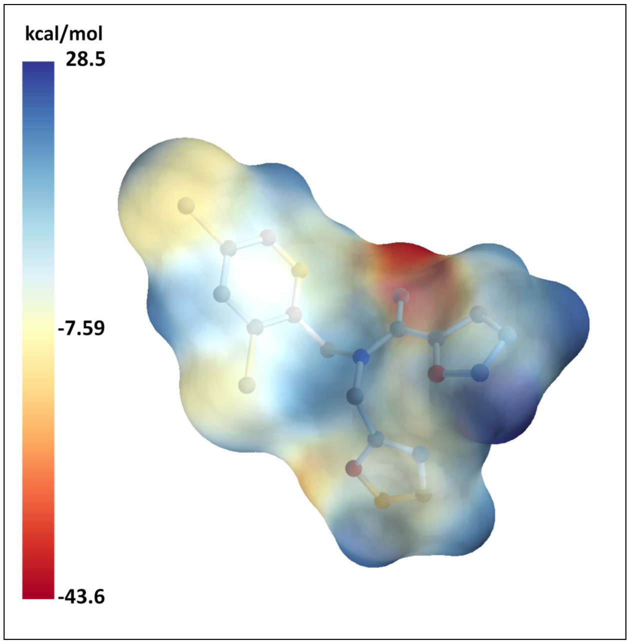

Fig. S1. Example of a molecular surface (electrostatic potential energy - kcal/mol) generated by the ESP-DNN program using a Neural Network for emulating the Quantum Mechanics calculations for molecule ZINC28231927.

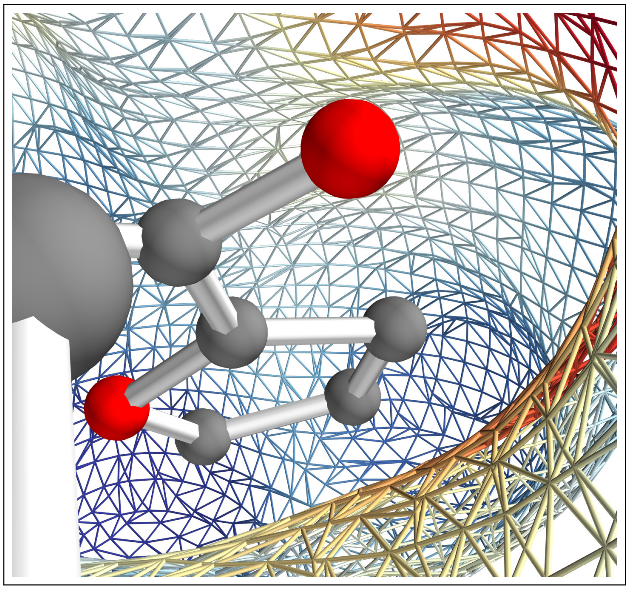

Fig. S2. Interior of a molecular mesh generated by the ESP-DNN program.

B. Surface Integration

As the mesh description for the surface and its electrostatic potential is not a continuous function of θ and ϕ, it is possible to evaluate equation (4) through integration via Surface Sampling Points (2, 3). Equation (4) can thus be rewritten as:

$$a_{\ell m} \approx \sum_{i=1}^{N} f(i)\, Y_{\ell}^{m}(i)\, \Delta\Omega(i)\,. \tag{S1}$$

We sample $i = 1 \ldots N$ (typically 10,000) points regularly distributed over the Unit Sphere and, in this case, the approximate element of surface area $d\Omega \approx \Delta\Omega(i) = \frac{4\pi r^2}{N}$ is just $\frac{4\pi}{N}$. Since the i sampling points are not necessarily the same as the mesh vertices, we employ the Möller–Trumbore intersection algorithm to find which triangle the associated ray intersects [4]. The intersection point, for tiny triangles, is where the projected sampling point touches the MLS. The value for the electrostatic potential at this position can be evaluated by an interpolation of the 3D triangle using barycentric coordinates of the projected sampling point relative to the vertices in which electrostatic values are calculated (fig. S3).

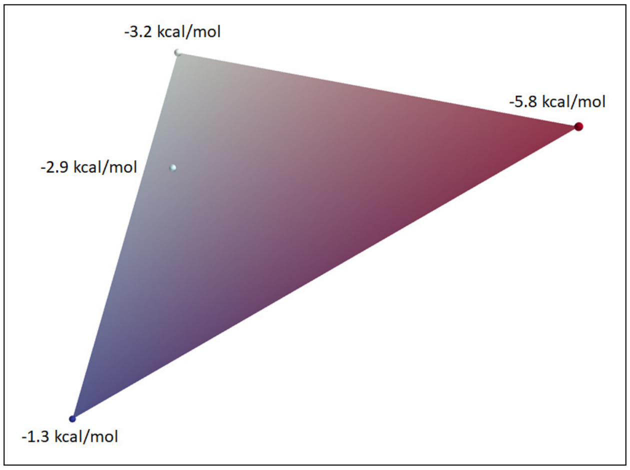

Fig. S3. Möller–Trumbor projected intersection and barycentric interpolation of a single spherical sampling.

C. Alignment of MLS via FFTs on the $SO(3)$ Rotation Group

In addition, LiraSearch allows us to compare all $(\ell_{max} + 1)^2 = 289$ SH coefficients for shape or electrostatics. As these SH coefficients are not rotationally invariant, a reproducible and efficient method is necessary to rotate them to align two shapes. The alignment is achieved through the implementation of the ICP algorithm [5] by Funkhouser *et al* [6] using the SOFT 1.0 SH library [7]. This technique determines the Wigner-D rotation matrix that aligns the two sets of SH coefficients by calculating an FFT on the $SO(3)$ Rotation Group. Given two surfaces with reconstructed functions $f(\theta, \phi)$ and $h(\theta, \phi)$, the correlation function $C(g)$ between them is:

$$C(g) = \int_{S^2} f(\theta, \phi)\overline{\Lambda(g)h(\theta, \phi)}d\Omega, \tag{S2}$$

where $g \in SO(3)$ and $\Lambda(g)$ is the linear operator that produces a Wigner-D function when applied to Y_l^m. Essentially, the algorithm finds the g that maximises the correlation function (eq. S2) between the two shapes described by $f(\theta, \phi)$ and $h(\theta, \phi)$ (eq. 1). By applying the optimal Wigner-D rotation matrix to the SH coefficients of one of the functions, the algorithm aligns both shapes into an orientation where the direct comparison of all coefficients is possible.

2. LIRASEARCH BENCHMARKING AGAINST DUDE-Z

A. Benchmark Protocol

The benchmarking was conducted using the DUDE-Z dataset [8], a widely used benchmark for ligand-based virtual screening that comprises 43 protein targets, each associated with a set of known active compounds and a larger set of property-matched decoys. Conformer generation followed the same protocol as the ROSHAMBO2 study [9]: up to 60 conformers per compound were generated with the ETKDGv3 distance-geometry method [10], followed by energy minimisation with the MMFF94s force field [11]. Compounds that failed embedding entirely were excluded from scoring. For each target, a leave-one-out (LOO) cross-validation protocol was applied: each active compound in turn was used as the query molecule, and the remaining actives together with all decoys for that target were ranked by similarity to the query. This procedure was repeated for

every active compound in the target family, and the results were aggregated to produce a single enrichment curve per target.

B. Enrichment Factor Calculation

The enrichment factor at 1% (EF1%) is the primary metric used to assess early enrichment performance. It quantifies the ratio of active compounds recovered in the top 1% of the ranked library relative to what would be expected by random selection, and is defined as:

$$\mathrm{EF}_{1\%} = \frac{N_{\text{actives,top1\%}} / N_{\text{top1\%}}}{N_{\text{actives,total}} / N_{\text{total}}} \tag{S3}$$

where $N_{\text{actives,top1\%}}$ is the number of actives recovered in the top 1% of the ranked list, $N_{\text{top1\%}}$ is the total number of compounds in the top 1%, $N_{\text{actives,total}}$ is the total number of actives, and N_{total} is the total number of compounds in the library (actives and decoys combined). An EF1% value of 1 corresponds to random performance, while the theoretical maximum is $100/1 = 100$, achieved when all top-ranked compounds are actives. The reported EF1% for each target is the mean value over all LOO queries performed for that target.

C. Methods Compared

Two modes of ROSHAMBO2 are compared against LiraSearch: shape-only (*shape*) and combined shape and pharmacophore (*combination*) [9]. ROSHAMBO2 is a GPU-accelerated ligand similarity tool that employs Gaussian-based shape and pharmacophore alignment, delivering over 200-fold performance improvements over earlier implementations. The shape-only mode optimises alignment based solely on molecular shape overlap, whereas the combination mode additionally incorporates pharmacophoric constraints during alignment.

LiraSearch was evaluated using its combined shape and electrostatic potential (ESP) descriptor mode, in which molecular surfaces are encoded as spherical harmonic expansions of both the surface geometry and the electrostatic potential predicted by the graph-convolutional neural network of Rathi *et al.* [1]. Unlike ROSHAMBO2, LiraSearch does not require explicit molecular alignment during the initial screening stage; instead, it exploits the rotational invariance of the spherical harmonic descriptor to enable alignment-free similarity comparisons, with optional FFT-based alignment available for post-screening superposition (Appendix 1.C).

D. Results

Results for all 43 DUDE-Z targets are reported in figure S4, and table S1. LiraSearch achieved a mean $\mathrm{EF}_{1\%}$ of 7.67 and a median of 6.81 across the 43 targets (standard deviation 6.32). Thirty-seven targets (86%) exceeded the random-selection baseline ($\mathrm{EF}_{1\%} > 1$), and 26 targets (60%) exceeded the twice-random threshold ($\mathrm{EF}_{1\%} > 2$). Eleven targets (26%) achieved $\mathrm{EF}_{1\%} > 10$, with FABP4 reaching the highest value ($\mathrm{EF}_{1\%} = 28.52$), followed by HS90A (20.46), MAPK2 (19.40), NRAM (17.37), FGFR1 (16.46), and DEF (15.08). Two targets, EGFR and FA10, returned $\mathrm{EF}_{1\%} = 0$. The mean AUC-ROC was 0.563 (median 0.566).

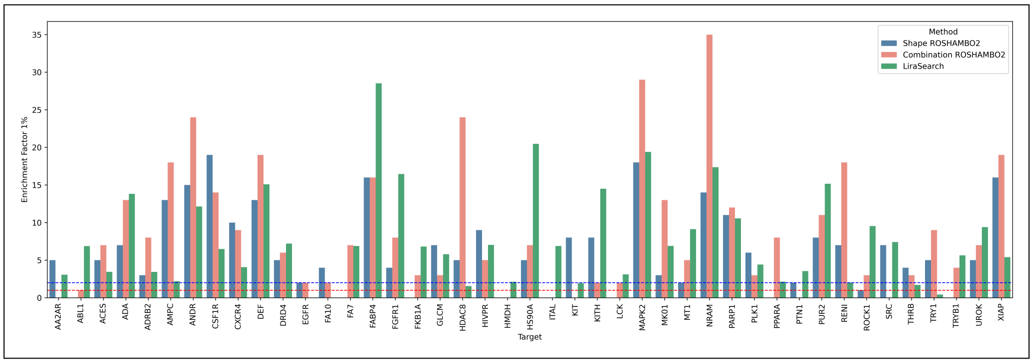

Fig. S4. Early enrichment factors at 1% for each of the 43 DUDE-Z targets using two alignment strategies in ROSHAMBO2 (shape-only optimization in blue, and combined shape and color optimization in red), and LiraSearch (green)

Table S1. USRCAT virtual screening performance on the 43 DUDE-Z targets. $EF_{1\%}$: early enrichment factor at 1% of the ranked dataset. AUC: area under the ROC curve. N_{act} and N_{dec}: number of active and decoy compounds per target after exclusion of embedding failures. Targets are sorted alphabetically.

**Target**	N_{act}	N_{dec}	$EF_{1\%}$	AUC
AA2AR	2610	129904	3.06	0.609
ABL1	1470	89104	6.87	0.530
ACES	840	71040	3.45	0.459
ADA	1800	95303	13.82	0.634
ADRB2	960	69240	3.44	0.477
AMPC	1410	90713	2.20	0.625
ANDR	840	59500	12.13	0.752
CSF1R	1620	100433	6.48	0.556
CXCR4	810	56899	4.07	0.691
DEF	1530	78896	15.08	0.628
DRD4	540	39589	7.21	0.566
EGFR	2040	118402	0.00	0.418
FA10	600	35549	0.00	0.263
FA7	930	59757	6.88	0.459
FABP4	1170	43380	28.52	0.729
FGFR1	1050	68879	16.46	0.673
FKB1A	1350	67230	6.81	0.714
GLCM	899	59184	5.78	0.587
HDAC8	2250	118799	1.55	0.388
HIVPR	810	74159	7.03	0.472
HMDH	990	54900	2.12	0.586
HS90A	1440	85168	20.46	0.665
ITAL	870	59548	6.89	0.646
KIT	1350	74309	1.92	0.564
KITH	1710	78553	14.50	0.594
LCK	1800	102543	3.11	0.497
MAPK2	2220	110394	19.40	0.736
MK01	1290	62458	6.89	0.524
MT1	1140	53436	9.12	0.463
NRAM	2430	158363	17.37	0.768
PARP1	2700	162756	10.55	0.619
PLK1	1290	63059	4.42	0.503
PPARA	840	17100	2.14	0.445
PTN1	2340	120207	3.55	0.546
PUR2	1200	31800	15.17	0.747
RENI	2310	93635	2.03	0.571
ROCK1	870	57719	9.54	0.573
SRC	1620	97254	7.41	0.504
THRB	1170	70289	1.71	0.375
TRY1	1410	85478	0.43	0.356
TRYB1	1080	51745	5.64	0.499
UROK	2160	118525	9.40	0.565
XIAP	2130	92548	5.40	0.646
**Mean**			**7.67**	**0.563**
**Median**			**6.81**	**0.566**

LiraSearch achieves competitive early enrichment performance compared with ROSHAMBO2 for the majority of targets. It outperforms both ROSHAMBO2 modes on several targets, including FABP4 (28.52 vs. 15.82/16.11), HS90A (20.46 vs. 5.2/7.36), FGFR1 (16.46 vs. 3.98/8.21), and PUR2 (15.17 vs. 7.79/11.21), demonstrating the complementary value of quantum-mechanically grounded electrostatic descriptors in recovering structurally diverse actives. Conversely, for targets such as HDAC8, NRAM, and RENI, the ROSHAMBO2 combination mode yields higher EF1% values, reflecting the strengths of pharmacophore-based alignment for those target families. Targets with low EF1% across all methods, including EGFR, FA10, and TRY1, suggest that these families present inherent difficulty for shape- and electrostatics-based similarity searching regardless of the specific method employed. EF1% measures early enrichment at the top of the ranked list, reflecting operational utility in a prospective screen. In contrast, AUC-ROC (table S1) summarises global discrimination across all score thresholds. These two metrics can diverge substantially, which makes both necessary for a comprehensive assessment of virtual screening performance.

REFERENCES

1. P. C. Rathi, R. F. Ludlow, and M. L. Verdonk, "Practical high-quality electrostatic potential surfaces for drug discovery using a graph-convolutional deep neural network," J. Medicinal Chem. **63**, 8778–8790 (2020). PMID: 31553186.
2. D. Ritchie and G. J. L. Kemp, "Fast computation, rotation, and comparison of low resolution spherical harmonic molecular surfaces," J. Comput. Chem. **20** (1999).
3. R. Green, "Spherical harmonic lighting: The gritty details," (2003).
4. C. Schlick and G. Subrenat, "Ray intersection of tessellated surfaces: Quadrangles versus triangles," in *in Graphics Gems,* (Academic Press, 1993), pp. 232–241.
5. P. Besl and N. D. McKay, "A method for registration of 3-d shapes," IEEE Transactions on Pattern Analysis Mach. Intell. **14**, 239–256 (1992).
6. T. Funkhouser, M. Kazhdan, P. Shilane, *et al.*, "Modeling by example," ACM Trans. Graph. **23**, 652–663 (2004).
7. P. J. Kostelec and D. N. Rockmore, "Ffts on the rotation group," in *Santa Fe Institute Working Papers Series Paper,* (2003), pp. 03–11.
8. R. M. Stein, Y. Yang, T. E. Balius, *et al.*, "Property-unmatched decoys in docking benchmarks," J. Chem. Inf. Model. **61**, 699–714 (2021). PMID: 33494610.
9. R. Atwi, S. Farr, Y. Wang, *et al.*, "Roshambo2: Accelerating molecular alignment for large chemical libraries with gpu optimization and algorithmic advances," J. Chem. Inf. Model. **65**, 9842–9849 (2025).
10. S. Riniker and G. A. Landrum, "Better informed distance geometry: Using what we know to improve conformation generation," J. Chem. Inf. Model. **55**, 2562–2574 (2015). PMID: 26575315.
11. T. A. Halgren, "Mmff vi. mmff94s option for energy minimization studies," J. Comput. Chem. **20**, 720–729 (1999).
